# A two-year follow-up of asfotase alfa replacement in a patient with hypophosphatasia: clinical, biochemical, and radiological evaluation

**DOI:** 10.20945/2359-3997000000222

**Published:** 2020-03-30

**Authors:** Fernanda Salles Reis, Débora Cristiane Gomes, Henrique Pierotti Arantes, Marise Lazaretti-Castro

**Affiliations:** 1 Departamento de Medicina Universidade Federal de São Paulo São Paulo SP Brasil Departamento de Medicina, Disciplina de Endocrinologia, Universidade Federal de São Paulo (Unifesp), São Paulo, SP, Brasil; 2 Departamento de Medicina Serviço de Endocrinologia Pediátrica Universidade Federal de Uberlândia Minas Gerais MG Brasil Departamento de Medicina, Serviço de Endocrinologia Pediátrica, Universidade Federal de Uberlândia, Minas Gerais, MG, Brasil; 3 Instituto Master de Ensino Presidente Antônio Carlos Araguari MG Brasil Instituto Master de Ensino Presidente Antônio Carlos (IMEPAC), Araguari, MG, Brasil

## Abstract

Hypophosphatasia (HPP) is a rare disease with a high mortality rate in its severe forms. It is caused by mutations within the gene encoding the tissue-nonspecific alkaline phosphatase (TNSALP), an enzyme responsible for bone mineralization. In 2015, the Food and Drug Administration approved the use of asfotase alfa, the first medication showing benefit in the treatment of HPP. We describe a case with a 2-year follow-up of the first Brazilian child treated with asfotase alfa. A 5-year-old boy, born to consanguineous parents, was diagnosed with HPP at the age of 20 months. During prenatal ultrasonography, polyhydramnios and shortening of long bones were detected. After birth, he presented delayed motor development, repeated respiratory infections, and bone deformities. At the age of 2 years and 8 months, he started walking and had already lost his primary teeth. He had reduced levels of alkaline phosphatase (ALP), elevated levels of pyridoxal 5’-phosphate (PLP), and a p.Ala33Val (c.98C>T) missense mutation in homozygosis in the TNSALP gene. His parents and sister also had reduced ALP levels, high PLP levels, and the same mutation in heterozygosis. His father and sister were healthy, and his mother was diagnosed with rickets in childhood, which resulted in short physical stature and lower limb deformities. The patient was started on asfotase alfa at the age of 2 years and 10 months. After 2 years of treatment, he improved his motor skills, had no further episodes of severe respiratory infection, and showed improved radiological findings of rickets, without any severe side effect.

## INTRODUCTION

Hypophosphatasia (HPP) is an inborn error of metabolism caused by a mutation within the gene that encodes the tissue-nonspecific alkaline phosphatase (TNSALP), an enzyme present in several tissues like bone, liver, and kidney (1). Alkaline phosphatase (ALP) is a hydrolase that removes phosphate groups from a large number of molecules. A reduction in ALP activity leads to an accumulation of three main substrates, namely, phosphoethanolamine (PEA), pyridoxal 5’-phosphate (PLP), and inorganic pyrophosphate (PPi) (2,3).

PPi inhibits hydroxyapatite crystals formation and growth, causing rickets, osteomalacia, and deposition of calcium pyrophosphate crystals in the joints (2). PLP is the major circulating form of vitamin B6. TNSALP dephosphorylates PLP into pyridoxal, enabling this form of the vitamin to cross the blood-brain barrier. Intracellular pyridoxal is rephosphorylated to function as a cofactor for enzymatic reactions such as the formation of gamma-aminobutyric acid (GABA), an inhibitory neurotransmitter. Therefore, TNSALP deficiency can lead to PLP accumulation in blood (which can be measured as vitamin B6) and to pyridoxine-dependent seizures (1,2).

The clinical manifestations of HPP vary substantially, ranging from neonatal death to premature tooth loss, short stature, or nonspecific symptoms like bone pain and reduced muscle strength. In addition to the symptoms mentioned above, HPP should be investigated in the presence of bone demineralization, bone deformities suggestive of rickets, craniosynostosis, recurrent fractures, pulmonary hypoplasia, myopathy, and delayed motor development (4).

Laboratory findings helpful in diagnosing HPP include reduced serum ALP levels and high serum PLP and urinary PEA levels. Interpretation of ALP results must take into account the age- and sex-related reference values for the method (5). HPP is still poorly recognized because the lower limit of ALP is often neglected (6). In situations where ALP levels are low, high vitamin B6 levels complement the diagnosis of HPP. Measurement of PPi and PEA levels may be helpful when the diagnosis of HPP is unclear, but these tests are still not commercially available in Brazil.

HPP treatment consists of enzyme replacement therapy (7). In 2015, regulatory agencies in Japan, Canada, the United States, and the European Union approved the use of asfotase alfa, a modified copy of the human ALP enzyme produced by recombinant DNA. Before asfotase alfa, HPP treatment was palliative and had high morbidity and mortality (8). This is the first medication to show benefit in the treatment of HPP and was approved in Brazil in 2017.

We present here the first child with HPP treated with asfotase alfa in Brazil, including a 2-year follow-up.

## CASE REPORT

The patient was a 5-year-old boy from Minas Gerais (Brazil), born from consanguineous parents. At 27 weeks of gestation, an obstetric ultrasonography detected polyhydramnios and femoral and humeral length shortening, with the length of all long bones below the 5th percentile for gestational age. He was born at full term by cesarean delivery, had Apgar scores of 9 and 10 at 1 and 5 minutes, respectively, weighed 3.3 kg, and measured 46 cm in length.

The patient had delayed motor development since birth. At the ages of 10 and 11 months, he was diagnosed with severe pneumonia, which required intensive care unit admission and mechanical ventilation. He was hospitalized on five other occasions due to pneumonia and was treated with oxygen therapy via mask or nasal cannula. His deciduous teeth erupted when he was 16 months old, but started to fall out shortly thereafter, and during evaluation, he only had two molar teeth. The patient only started walking with support at the age of 2 years and 8 months.

**Figure 1:**
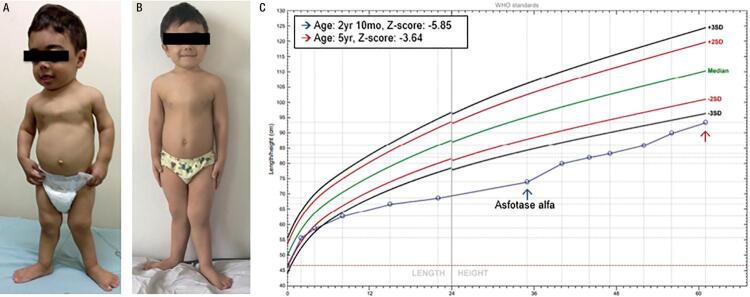
Progression of the patient during 2 years of follow-up. (A) Before treatment (2 years 10 months). (B) After 2 years of treatment (5 years). (C) Sex- and age-specific length/height chart (World Health Organization).

**Figure 2:**
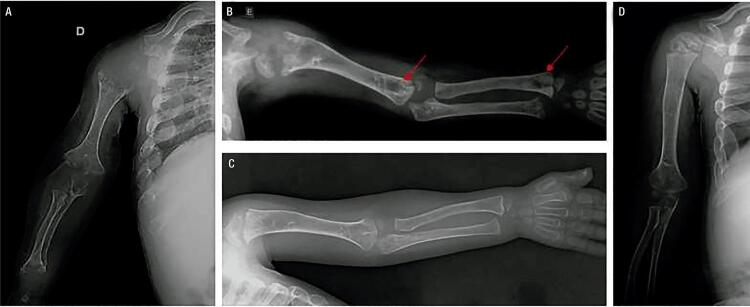
Upper limb radiographs. (A) Age 1 year and 2 months: diffuse bone hypomineralization, cupping, fraying and widening metaphyses, thin cortical bone, and diaphyseal shortening. (B) Age 2 years and 10 months, prior to treatment: metaphyseal “tongues” of radiolucency (arrows). (C) Age 4 years, after 1 year of treatment. (D) Age 5 years, after 2 years of treatment: improvement of the signs of rickets. Treatment improved bone mineralization and formation, cupping, fraying and widening metaphyses, and radiolucent “tongues”. The long bone became better defined, and the lytic and sclerotic metaphyseal areas resolved.

**Figure 3:**
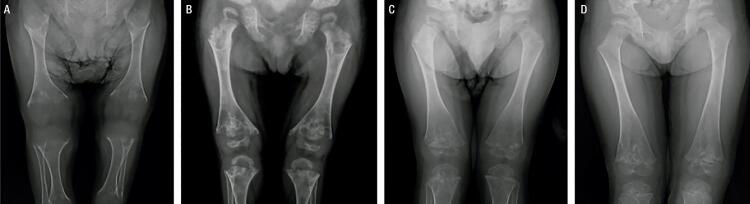
Lower limb radiographs. (A) Age 1 year and 2 months, prior to treatment. (B) Age 2 years and 10 months, just prior to treatment with asfotase alfa. (C) Age 4 years, after 1 year of treatment. (D) Age 5 years, after 2 years of treatment. Improvements similar to those in the upper limbs also occurred in the lower limbs.

**Figure 4:**
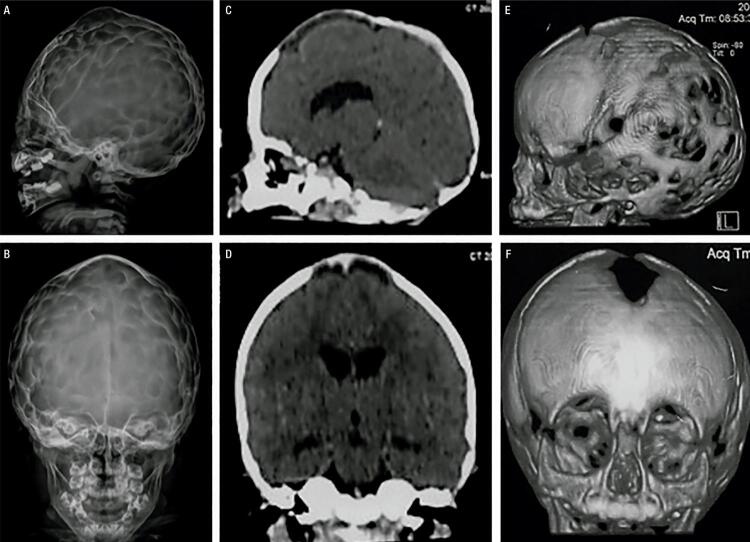
Skull images at the age of 1 year and 8 months. (A-B) Radiographs showing the “beaten copper sign”. (C-F) Cranial computed tomography (CT) images showing misshapen skull, digitiform impressions, and prominence of the brain parenchyma at the anterior fontanelle.

He was diagnosed with HPP at the age of 20 months. His laboratory tests revealed a low ALP (18 U/L, normal range for age and sex [NR]: 104-345 U/L), high vitamin B6 (250 µg/L; NR: 5.2-34 µg/L), and total calcium within the upper limit of normal (10.8 mg/dL, NR: 8.8-10.8 mg/dL). Other blood parameters were normal (Table 1). On physical exam, he presented proptotic eyes, misshapen skull, chest deformity, rachitic rosary, thoracolumbar kyphosis, short stature, short limbs, and knock knees (Figure 1A). Radiographs revealed diffuse bone hypomineralization, cupping, fraying and widening metaphyses, metaphyseal “tongues” of radiolucency (areas of unmineralized bone), thin cortical bone, thin ribs, shortening of long bones’ diaphyses, and copper beaten skull (Figures 2A-B and 3A-B). Cranial computed tomography showed digitiform impressions, craniocerebral disproportion, and prominence of the brain parenchyma at the anterior fontanelle (Figure 4).

**Table 1:** Laboratory tests at diagnosis and during follow-up

Laboratory tests	At diagnosis	1 year of treatment	2 years of treatment
Alkaline phosphatase (NR: 104-345 U/L)	18	5161	423
Total calcium (NR: 8.8-10.8 mg/dL)	10.8	9.9	10
Phosphorus (NR: according to age, mg/dL)	5.4 (4.5-6.7)	6.39 (4.5-5.5)	5.9 (3.3-5.6)
Parathyroid hormone (NR: 4-58 pg/mL)	12	8	12
Creatinine, serum (NR: 0.3-0.7 mg/dL)	0.4	0.32	0.4
25-hydroxyvitamin D (NR: >20 ng/mL)	39.5	39.3	25.9
24-h urinary calcium (NR: up to 4 mg/kg/24h)	21.9 (3 mg/kg/24h)	-	-

NR: normal range.

His parents and sister also had reduced ALP and increased PLP levels. His mother (36 years old) was diagnosed with rickets in childhood due to lower limb deformity and received no diagnosis of a specific cause or appropriate treatment. She had a stature of 1.44 m (target height 1.55 m), chronic pain, bowing legs (*genu varum)*, ALP 16 U/L (NR for age: 30-120 U/L), and vitamin B6 65.8 µg/L (NR: 5.2-34 µg/L). She underwent dual-energy X-ray absorptiometry, which showed normal bone density, and histomorphometric analysis of a non-decalcified bone biopsy sample, which showed low remodeling but no signs of osteomalacia. His father (36 years old) and sister (8 years and 7 months old) had no history of fractures, bone deformity, or other clinical abnormalities observed until the publication of this article. The father’s laboratory tests showed ALP 28 U/L (NR for age: 30-120 U/L) and vitamin B6 151 µg/L (NR: 5.2-34 µg/L). The patient’s sister underwent annual routine blood tests, and at the age of 8 years and 4 months, she presented low ALP level 141 U/L (NR for age and sex: 142-335 U/L) and vitamin B6 86 µg/L (NR: 8.7-27.2 µg/L).

A genetic analysis of the patient, performed by Mendelics (São Paulo, Brazil), identified a homozygous c.98C>T (p.Ala33Val) missense mutation in the *TNSALP *gene. The same mutation was found in heterozygosis in the patient’s parents and sister. This mutation has already been described in association with HPP (7,9).

### Treatment with asfotase alfa

The patient started treatment with subcutaneous asfotase alfa 2 mg/kg thrice weekly at the age of 2 years and 10 months. At that time, his height was 74 cm (Z-score -5.85), and his weight was 9.5 kg (Z-score -3.3). After treatment initiation, he no longer required hospitalization due to pneumonia. He improved his gait speed and pattern and started to run after 6 months of treatment. The 6-minute walk test was not performed because the patient was unable to follow properly our instructions. His growth rate improved to a growth velocity of 10 cm/year. At the age of 5 years, his height was 93.5 cm (Z-score -3.64) and his weight was 15.5 kg (Z-score -1.32) (Figures 1B and C). Until the publication of this article, the patient had presented no reaction at the injection site. During the treatment period, he underwent correction of craniosynostosis, without complications.

His biochemical studies over the course of therapy showed an increase in serum phosphorus (5.9 mg/dL; NR: 3.3-5.6 mg/dL), while serum calcium and PTH remained normal. ALP levels increased due to the medication (Table 1). Radiographs showed improvement of rickets signs (Figures 2C-D and 3C-D). A panoramic dental X-ray showed tooth eruption with little or no root development.

This case report was approved by the institution’s Ethics Committee (CAAE 77107817.2.0000.5505), and a written informed consent was obtained from the patient’s parents.

## DISCUSSION

HPP is classified into clinical forms according to the age of onset of the clinical manifestations. Perinatal HPP is the most severe form of the disease, manifesting prenatally with profound bone demineralization (10,11). A benign prenatal form is characterized by skeletal abnormalities on prenatal ultrasonography, followed by a mild to moderate postnatal clinical course (1). In infantile HPP, the manifestations begin in the first 6 months of life, and the patients may present anorexia, failure to thrive, hypotonia, delayed motor development, craniosynostosis, pyridoxine-dependent seizures, and clinical and radiological signs of rickets (7,10,12). Childhood HPP occurs after the age of 6 months with a heterogeneous presentation, ranging from mild to severe symptoms, including typical rachitic deformities, short stature, premature loss of dentition, bone pain, and muscle weakness (13,14). Adult HPP is characterized by chondrocalcinosis, muscle weakness, and increased risk of fractures (15,16). Odontohypophosphatasia is the mildest form of the disease, manifesting at any age with early tooth loss (17).

We reported here a case of a patient with a benign prenatal form of HPP treated with asfotase alfa for 2 years, with great improvement on his clinical features. Although the diagnosis was established at the age of 1 year and 8 months, the first evidence of bone deformation was identified prenatally on gestational ultrasonography, defining the prenatal form of the disease.

The clinical presentation of our patient was characterized mainly by bone changes. His mutation (c.98C> T) was first described in 1992 in a patient with infantile HPP (9). Recently, the same homozygous mutation was described in a female patient with clinical manifestations similar to those of our patient (7). We will have the opportunity to follow up on our patient with enzyme replacement during his growth. The accumulation of information about potential benefits and side effects of this new treatment in a real-life setting, outside of controlled studies, will be fundamental. Freitas and cols. described another Brazilian patient who also presented with pediatric-onset HPP and was treated with asfotase alfa for 1 year. However, because the diagnosis of this patient was only established later in adulthood, the patient had severe and probably irreversible skeletal deformities (18).

Incomplete penetrance (proportion of individuals with clinical disease among individuals with the same genotype) of *TNSALP* mutations has already been described (19) and can be confirmed in our patient by the clinical manifestations of the patient’s relatives. Although his parents and sister had the same mutation in heterozygosis, only his mother presented clinical manifestations to date.

Asfotase alfa is the first medication approved for the treatment of HPP. It is currently indicated for patients at any age with pediatric-onset HPP to treat the bone manifestations of the disease (20). The first study showing the benefit of asfotase alfa treatment in humans was published in 2012 and evaluated 11 children with severe HPP forms. Asfotase alfa improved the patients’ overall survival, muscle strength, motor and respiratory function, bone pain, bone mineralization, and radiographic signs of rickets (7). Historical data show 1-year and 5-year survival rates of untreated patients as 42% and 27%, respectively. Asfotase alfa treatment increased the survival rates to 95% and 84% at 1 and 5 years, respectively (21). With treatment discontinuation, the signs and symptoms of rickets return quickly (22). The patient’s serum ALP increased with treatment and remained greater than 1,000 U/L in most measurements. This significant increase in ALP has been reported with treatment and has not been associated with harmful effects to date (23).

The patient’s parents and sister also have HPP and are currently being monitored for clinical manifestations. Data showing the benefits of treating mild and late forms of HPP are still limited. However, precise and early diagnosis of HPP is fundamental, since medications widely used in bone diseases, like bisphosphonates and calcium salts, are contraindicated in this condition. Bisphosphonates may increase the risk of atypical femoral fractures, and calcium salts can worsen nephrocalcinosis (23,24). Only in rare situations, such as in hypocalcemia during enzyme replacement therapy, calcium salts can be used in patients with HPP (23).

The most common side effects of asfotase alfa include injection site reactions, which are usually mild, such as erythema, pruritus, nodule, and pain. Other adverse reactions included headache, myalgia, irritability, pyrexia, hypocalcemia, hyperphosphatemia, lipodystrophy, and ectopic calcifications (20,23,25). During treatment, our patient developed hyperphosphatemia, and his craniosynostosis worsened. Ectopic calcification and craniosynostosis are known manifestations of HPP that seem to not improve with asfotase alfa treatment. In fact, it is unclear whether the treatment could worsen the progression of these manifestations. Data available to date are insufficient to establish any relationship in this regard (20).

In summary, we described the characteristics of a patient with HPP carrying a homozygous mutation in the *TNSALP* gene and his 2-year follow-up during treatment with asfotase alfa. This is the first child with HPP treated with asfotase alfa in Brazil, showing significant clinical and radiological improvements with the medication. HPP has a wide spectrum of clinical presentations, and the most severe cases are seen in homozygous forms of the disease. However, milder and later manifestations can be observed in heterozygous carriers. Asfotase alfa has changed the prognosis of HPP by increasing the survival and quality of life of patients affected by the disease.
